# Phase Ia study to evaluate RO7497987, a FLT3L-fragment Fc fusion protein, in healthy volunteers

**DOI:** 10.3389/fimmu.2026.1784546

**Published:** 2026-03-24

**Authors:** Anthony J. Iacovelli, Shomyseh Sanjabi, Sharareh Monemi, Assaf Amitai, Jérémie Decalf, Christine Moussion, Michael Z. Liao, Gautham K. Rao, Jennifer Getz, Michael R. Mancuso

**Affiliations:** Genentech, Inc., South San Francisco, CA, United States

**Keywords:** clinical trial, dendritic cells, FLT3L-Fc, Fms-related tyrosine kinase 3 ligand, RO7497987

## Abstract

**Background:**

Dendritic cells play a critical role in immunity. Fms-related tyrosine kinase 3 ligand (FLT3L) is a cytokine that can promote the expansion and differentiation of bone marrow dendritic cells (DC) progenitors. RO7497987 (FLT3L-Fc) is a novel FLT3 agonist developed to extend the half-life of FLT3L and induce expansion of peripheral blood DC.

**Methods:**

This Phase Ia trial evaluated the safety, tolerability, pharmacokinetics, and pharmacodynamics of RO7497987 in healthy volunteers. Forty-four participants received RO7497987 as a single ascending dose (SAD, 0.7–70 mg) or multiple ascending doses (MAD, 7 or 21 mg, 21 days apart).

**Results:**

RO7497987 was safe and well-tolerated at all doses. The most common treatment-related adverse events (AE) were Grade 1 myalgia, headache, and lymphadenopathy, all of which resolved spontaneously. There were no Grade ≥ 3 AEs, serious AEs, or dose-limiting adverse events. Pharmacokinetic analysis showed dose-dependent increases in FLT3L-Fc C_max_ and AUC_inf_ with no accumulation in the MAD cohorts. RO7497987 administration resulted in dose-dependent expansion of monocytes, as well as expansion of all major DC subsets, including conventional and plasmacytoid DC, which was sustained with multiple doses.

**Conclusion:**

These findings aid the design of dosing regimens of RO7497987 as an immunomodulatory agent.

**Clinical trial registration:**

https://www.isrctn.com/ISRCTN92655801, identifier ISRCTN92655801.

## Introduction

Dendritic cells (DC) make up a diverse group of specialized antigen presenting cells (APCs) that regulate adaptive immune responses, and play crucial roles in immunity to cancer and tolerance (1). Activated DC are relatively rare in tissues, more particularly, in tumor tissue with an immunosuppressive microenvironment (1–3). In such cancer types, low DC content is considered to be a limiting factor in successful immunotherapies (4). Since DC play a critical role in priming anti-tumor T cell immunity, they thereby represent a promising therapeutic target for novel cancer immunotherapy (CIT) approaches (5).

Fms-related tyrosine kinase 3 ligand (FLT3L) is a soluble growth factor (cytokine) and an important regulator of hematopoiesis. FLT3L functions by agonizing the receptor-type tyrosine-protein kinase FLT3 which is expressed on DC progenitors in the bone marrow (6–10). It has been established that agonism of FLT3 signaling promotes DC proliferation, differentiation, and survival (11–17). FLT3L administration in nonclinical models expands conventional type 1 (cDC1) and type 2 (cDC2) dendritic cells, plasmacytoid DC (pDC), and other myeloid subsets in blood, lymphoid organs, and tumors (18–21).

FLT3L possesses the ability to promote the expansion and differentiation of bone marrow DC progenitors. Those expanded preDC may then populate peripheral tissues, including tumors, where they could finish their differentiation, acquire tumor-derived antigens and develop immunogenic activity upon exposure to maturation signals. Nonclinical and early clinical proof-of-concept studies show that FLT3L administration can provide enhanced efficacy when used in combination with other cancer therapies and CIT agents (22–25).

CDX-301, a recombinant human FLT3L (rHu-FLT3L), and GS-3583, a novel FLT3 agonistic Fc fusion, are FLT3 agonists that have been evaluated in clinical trials. These studies show effective expansion of DCs in healthy human volunteers and cancer patients and that the expanded DCs can be activated by TLR agonism and/or vaccination (17, 24, 26–28).

RO7497987 (FLT3L-Fc) is a novel FLT3 agonist developed to extend the half-life of FLT3L and thus decrease the clinical dosing frequency needed to achieve optimal DC expansion. RO7497987 consists of a wild-type truncated extracellular domain of human FLT3L and an effectorless Fc portion of human IgG1 isotype (NG2LH) which are genetically fused from the C-terminus of FLT3L to the N-terminus of the Fc at the upper hinge (29). In nonclinical toxicity studies, a single dose of RO7497987 induced marked expansion of peripheral blood cDC1, cDC2, and pDC cells showing robust pharmacodynamic (PD) response without any adverse findings, providing sufficient safety margins for clinical trials (30, 31).

## Materials and methods

### Study design and participants

This Phase Ia open-label dose-escalation study (ISRCTN.com registration number ISRCTN92655801) was designed to evaluate the safety (primary study objective), tolerability, pharmacokinetics (PK), and PD of RO7497987 in single and multiple ascending doses in healthy volunteers. Exploring the pharmacological effects of RO7497987 on expansion of peripheral DC was the key PD objective. The study also examined the immune response to RO7497987 and aimed to identify the recommended dose and schedule for Phase II studies in cancer patients.

Participants were enrolled between the age of 18–65 years with a minimum weight of 40 kg at screening (BMI 18–32 kg/m^2^), had adequate hematologic and end-organ function, and had no history of bone marrow disorders or alpha-gal allergy or syndrome.

### Procedures

In the single-agent dose escalation stage participants were given intravenous RO7497987 (supplied by Genentech, Inc., South San Francisco, CA) as a single dose (SAD) at 0.7–70 mg in 5 cohorts. Subsequent doses increased by no more than 3.3-fold guided by safety and real time PK, PD, and immunogenicity data as reviewed by the Safety Monitoring Committee (SMC). The first in human dose of 700 μg was based on a minimum pharmacologically active dose and was projected to induce an 8-fold expansion in peripheral blood cDC1. Participants enrolled in the multiple ascending dose (MAD) cohorts received 2 doses of RO7497987 at 7 or 21 mg dose levels 21 days apart. The initial MAD cohort dose was determined by the SMC based on SAD data, with escalation limited to the highest safely cleared SAD dose. For both stages the RO7497987 infusion period was 90 minutes and no pre-medication was required or recommended prior to administration. Dose-limiting adverse events (DLAE) were evaluated from the start of study treatment until 21 days after the last dose of study treatment in both the SAD and MAD cohorts.

### Safety assessment

The safety objective for this study was to evaluate the safety of RO7497987 on the basis of incidence and severity of adverse events (AE) according to NCI CTCAE v5.0. Safety was assessed at screening, during the treatment period(s), during follow-up, and at end of study. DLAEs were defined as any Grade ≥ 2 AEs and serious AEs (SAE) occurring within 21 days after the last dose of RO7497987 and which could not be clearly attributed to another etiology. Per the protocol-defined stopping rules, dosing and enrollment would be suspended to allow for SMC data review if there was a significant safety risk to other participants, if a clinically significant pattern of toxicity was apparent in multiple participants, or if ≥ 1 Grade ≥ 3 DLAEs or ≥ 2 Grade 2 DLAEs at a given dose level occur.

### Pharmacokinetic and immunogenicity assessments

Blood samples were taken for PK evaluations at screening, during the treatment period(s), during follow-up, and at end of study. A validated ELISA measured RO7497987 serum concentrations with a lower limit of quantification of 0.00313 μg/mL RO7497987. Serum PK of RO7497987 was summarized by estimating total exposure area under the concentration-time curve (AUC), T_max,_ C_max_, total clearance, volume of distribution at steady state, and terminal half-life. The numbers and proportions of anti-drug antibody (ADA)-positive participants and ADA-negative participants at baseline and after drug administration were analyzed using a validated bridging ELISA. Serum samples that confirmed positive for ADA were further tested in a domain characterization ELISA.

### Pharmacodynamic activity

The PD objective for this study was to characterize the profile of RO7497987 on the basis of the kinetics, magnitude of expansion, and contraction of myeloid cells including DC and monocytes, in the peripheral blood following administration of RO7497987. The PD analyses included all participants who completed the study with sufficient data to enable estimation of key parameters. Blood samples were collected on the same schedule as PK assessments, with another sample drawn at the 1 week scheduled clinic visit (Day 8). Flow cytometry was performed on Cyto-Chex fixed whole blood using the standard WBC Dual Platform and the A167 TBNK in WB assays as well as a custom A788 Myeloid DC Subset including activation markers in whole blood assay on a 5 laser Cytek Aurora at Q^2^ Solutions (IQVIA Laboratories) to calculate absolute DC and monocyte cell counts per milliliter of blood. Potential expansion of Tregs was not measured in this study, as the samples were collected in Cytochex tubes, which is not supportive of measuring intracellular molecules, such as Foxp3. A cloud-based analysis software named OMIQ was used for manual gating analysis and to generate pre-defined reportables.

### Statistical analysis

The sample size for this study was based on practical considerations and the dose-escalation rules described above and is not based on statistical power calculations. Six participants dosed with RO7497987 in each cohort were deemed sufficient to characterize the single-dose safety, tolerability, and available PK data of RO7497987. The safety analysis population consisted of all participants who received ≥ 1 dose of study drug, with participants grouped according to treatment received. The anticipated risks of RO7497987, which include IRRs, elevated liver enzymes, and lymphadenopathy and organ enlargement are not expected to be confounded by background events and do not require blinding and placebo control for identification, clarification, and attribution to RO7497987 in order to meet the study safety objective.

## Results

### Baseline demographics

From 30 Nov 2021 to 19 Jan 2023, 44 participants were enrolled across 7 cohorts (SAD 1-5, MAD 1-2) at 1 site in the United States. The duration of follow-up for SAD Cohorts 1–2 was 50 days, SAD Cohorts 3–5 was 85 days, and MAD Cohorts 1–2 was 106 days.

Baseline characteristics are shown in **Table 1**. The median age was 42 years (range 22-65), sex was balanced (52% male, 48% female), and most participants were white (75%). In the 7 mg SAD cohort (7 mg), 1 participant was dosed 0.674 mg in error. Overall, 43 out of 44 participants completed the study. One participant in the 70 mg SAD cohort 5 (RO7497987–70 mg) discontinued from the study due to withdrawal by the participant.

**Table 1:** Healthy participant demographics.

Cohort	SAD1 0.7 mg^a^	SAD2 2.1 mg	SAD3 7.0 mg	SAD4 21 mg	SAD5 70 mg	MAD1 7.0 mg	MAD2 21 mg
Participants treated	6	7 (1 participant replaced due to dose error)	6	6	7 (1 participant replaced after unrelated AE)	6	6
Number of doses	1	1	1	1	1	2 (q 21d)	2 (q 21d)
Age in years, median (range)	37 (25-60)	52 (25-60)	46 (28-61)	49 (24-64)	49 (31-62)	38 (35-65)	42 (22-65)
Sex	2M, 4F	5M (1 replaced), 2F	3M, 3F	3M, 3F	4M, 3F (1 replaced)	5M, 1F	1M, 5F
Race							
Black/African American	1	0	3	3	3	1	0
White	5	4	2	2	4	5	5
Hispanic/Latino	0	3	1	1	0	0	1

a One participant was dosed 0.674 mg in error.

### Safety

All AEs were Grade 1 except for 2 Grade 2 AEs of procedural pain and tooth abscess; both of which were assessed as unrelated to study treatment. Five TEAEs were reported in ≥ 2 participants (**Table 2**). Treatment-related AEs reported in ≥ 2 participants included myalgia (n=4; 9%), and headache and lymphadenopathy (each n=3; 7%). All treatment-related AEs were Grade 1 and resolved spontaneously. The study completed without any Grade ≥ 3 AEs, SAEs, dose-limiting AEs, or deaths. A maximum tolerated dose was not identified. Based on laboratory evaluation, dose-dependent increases in monocytes and leukocytes were seen across all cohorts, which was an expected PD effect of RO7497987. No abnormal trends were seen in vital signs or ECG.

**Table 2:** Summary of AEs reported in ≥ 2 participants receiving doses of 7–70 mg RO7497987.

AE preferred term	SAD1 0.7 mg (n=6)		SAD2 2.1 mg (n=6)		SAD3 7.0 mg (n=6)		SAD4 21 mg (n=6)		SAD5 70 mg (n=7)		MAD1 7.0 mg (n=6)		MAD2 21 mg (n=6)		Total (N = 43)	Total related (N = 43)
	Gr1	Gr2	Gr1	Gr2	Gr1	Gr2	Gr1	Gr2	Gr1	Gr2	Gr1	Gr2	Gr1	Gr2	All Grade	All Grade
Headache			1 (17%)										3 (50%)		4 (9%)	3 (7%)
Myalgia					1 (17%)		2 (33%)						1 (17%)		4 (9%)	4 (9%)
Covid-19	–	–	–	–	–	–	1 (17%)	–	1 (14%)	–	–	–	–	–	2 (5%)	0
Lymphadenopathy	–	–	–	–	–	–	–	–	–	–	1 (17%)	–	2 (33%)	–	3 (7%)	3 (7%)
Upper respiratory tract infection	–	–	–	–	–	–	2 (33%)	–	–	–	–	–	1 (17%)	–	3 (7%)	0

### Pharmacokinetics and immunogenicity analysis

**Figure 1:**
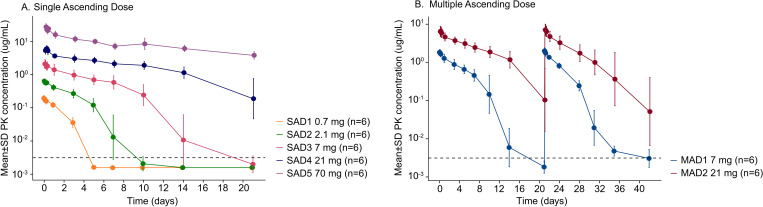
Serum concentration-time profiles of RO7497987 in **(A)** SAD and **(B)** MAD cohorts. One participant in SAD Cohort 5 was removed as samples were missing due to early termination. MAD cohorts (7 mg and 21 mg) received 2 doses of RO7497987 at the specified dose level 21 days apart by IV infusion. The black dashed line represents the lower limit of quantification (0.00313 ug/mL).

RO7497987 PK (**Figure 1**; **Supplementary Tables 1**, **2**) were well-characterized in healthy volunteers. There was a dose-dependent increase in FLT3L-Fc C_max_ and AUC_inf_ following RO7497987 administration; an approximately dose proportional increase with C_max_ and greater than dose proportional increase with AUC. In the 7 and 21 mg MAD cohorts, FLT3L-Fc C_max_ and AUC had no apparent accumulation after the second dose at Day 21. Increased FLT3L-Fc PK exposure correlated with increases in the profile of cDC expansion. Peak cDC1 expansion was achieved between 7–21 mg.

Five (17%) of the 30 tested participants in the SAD cohorts, and 1 (8%) out of 12 participants in the MAD cohorts, evaluable for ADA at any post-dose timepoint, developed ADA following dosing of RO7497987. Titer values ranged from 1.54-2.42 with a minimum reportable titer of 1.30.

One participant (SAD Cohort 5) had persistent ADAs, testing positive on Days 15, 22, 50, and 85. Five participants had transient ADAs, defined as an ADA positive result detected (a) at only 1 post-baseline sampling timepoint (excluding the last timepoint) OR (b) at ≥ 2 timepoints during treatment where the first and last ADA positive samples are separated by < 16 weeks, irrespective of any negative samples in between. Transient ADAs were detected in 4 SAD cohort participants (1 participant each in the 2.1 mg and 21 mg cohorts and 2 participants in the 70 mg cohort) and 1 participant in the 7 mg MAD cohort.

All confirmed ADA-positive samples were negative in the FLT3L characterization assay suggesting that the ADA were primarily directed against the Fc. No differences were observed in the PK, safety profiles, or PD measures in participants who developed ADA compared to those who did not (data not shown).

### Pharmacodynamic analysis

**Figure 2:**
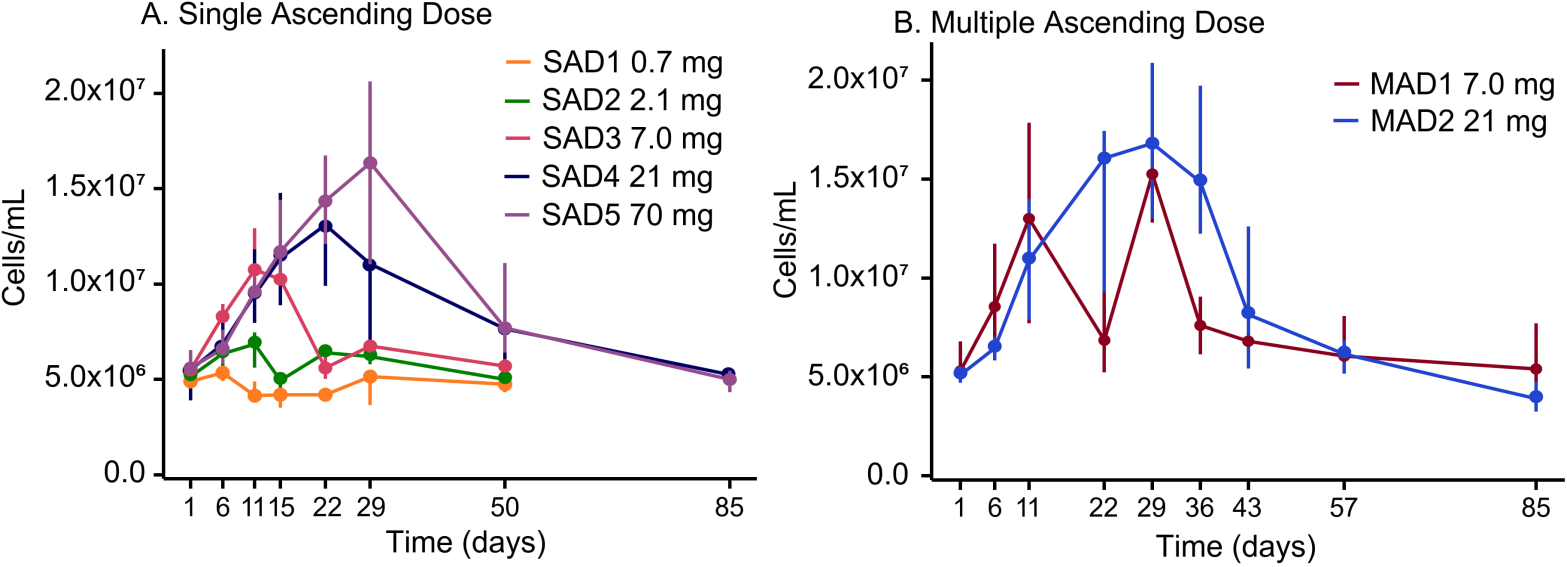
Total leukocyte counts across all **(A)** SAD and **(B)** MAD cohorts plotted as median values and IQR from 25th to 75th percentile.

**Figure 3:**
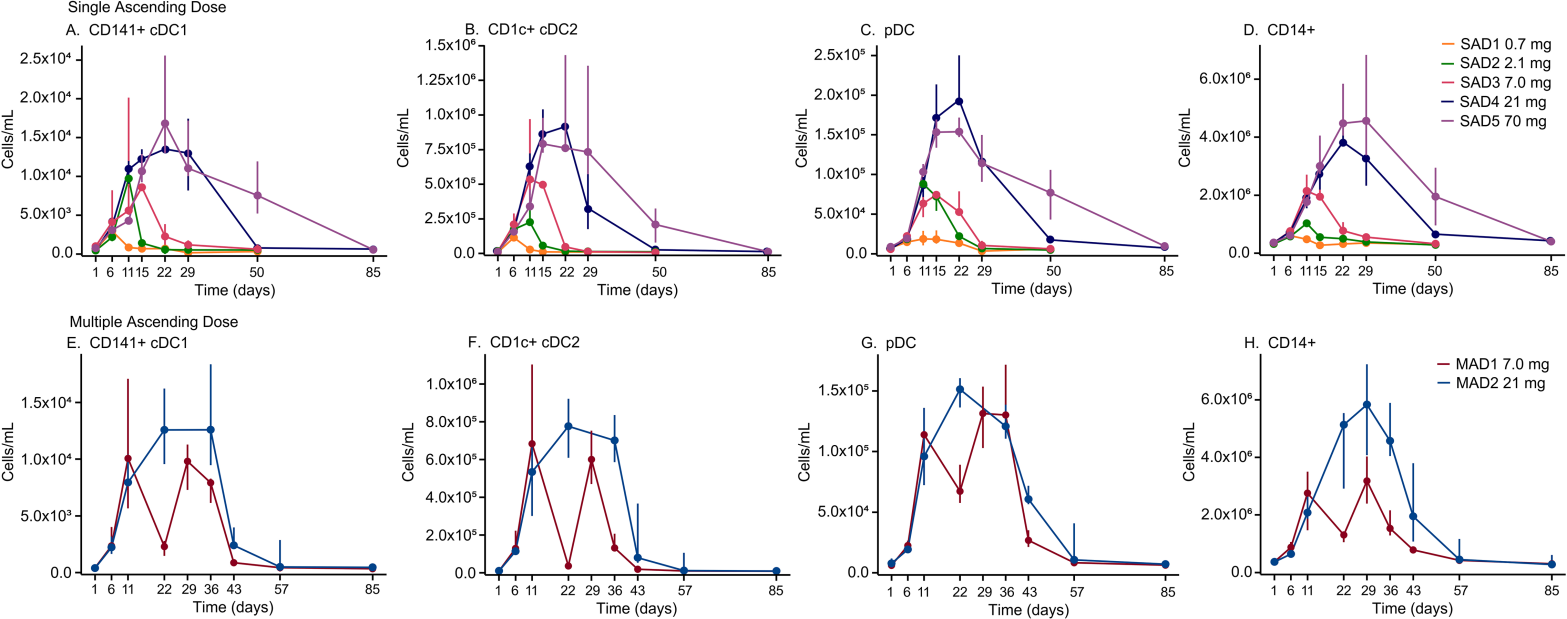
Profiles of dose-dependent expansion and maintenance of **(A, E)** cDC1, **(B, F)** cDC2, **(C, G)** pDC, and **(D, H)** CD14+ monocytes following RO7497987 administration. Data shown as median with upper and lower quartiles.

Administration of RO7497987 resulted in dose-dependent expansion of total leukocytes (**Figure 2**) primarily due to expansion of monocytes, and all major DC subsets, including cDC and pDC (**Figure 3**) with prolonged maintenance of these populations at higher doses. Single-dose administration of RO7497987 at 0.7 mg, 2.1 mg, 7.0 mg, 21 mg, and 70 mg led to dose-dependent changes in monocyte and DC populations. cDC1 and cDC2 showed robust and dose-dependent expansion across all dose levels. Lower doses (0.7 mg and 2.1 mg) resulted in modest increases in monocytes and pDC, with peaks occurring earlier and resolving more quickly. Higher doses (7.0 mg, 21 mg, and 70 mg) produced more substantial expansions of these cell types, with peaks appearing later and requiring more time to return to baseline.

Multiple doses of RO7497987 induced sustained expansion of monocyte and DC populations. Both the 7.0 mg and 21 mg cohorts exhibited peaks in monocyte and DC counts, with the higher dose generally showing a more prolonged elevation of these cells. Similarly, cDC1 and cDC2 displayed sustained and substantial expansion, with the 21 mg dose leading to a more persistent elevation compared to the 7.0 mg dose. Sustained increases in pDC was also shown in the multiple-dose cohorts. Several activation markers were included in the flow panel, including HLA-DR, CD40, CD80, and CD86 and none of these markers showed enhanced expression above baseline at all the evaluated time points.

## Discussion

This study was designed to evaluate the safety, tolerability, PK, and pharmacodynamic changes in immune cells after receipt of single and multiple ascending doses of RO7497987. In SAD and MAD cohorts, RO7497987 administration was safe and well-tolerated in healthy volunteers at the doses tested. We observed dose-dependent increases in FLT3L-Fc C_max_ and AUC with a longer half-life at higher doses, along with dose-dependent increases in DC and monocytes.

The single dose and multiple dose RO7497987 PK was well-characterized. Total clearance results suggest an impact of TMDD on PK at lower doses; consistent with the mechanism of action. Across the first doses of the 2 MAD cohorts, the PK was similar to that of the SAD cohorts and no apparent accumulation was observed after the second dose. mPBPK/PD model describes SAD and MAD data well, and simulations support 7 mg as a single dose administration, as well as 7 mg given every ≥ 4 weeks, if given as part of a cancer immunotherapy regimen where transient FLT3 agonism is desired (30, 31).

Prior clinical studies have shown that FLT3 agonists exhibited acceptable tolerability in healthy volunteers. These include recombinant FLT3L at doses up to 100 μg/kg/day for 14 days, CDX-301 (a soluble recombinant human FLT3L) at doses of 25 μg/kg/day for 7 or 10 days or 75 μg/kg/day for 5 days, and single doses of GS-3583 (a FLT3 agonist Fc fusion protein) up to 2,000 μg (12, 17, 27). In this study, RO7497987 demonstrated a favorable safety profile across all doses explored. Most participants experienced Grade 1 AEs, while 2 participants experienced Grade 2 AEs (not related to study treatment). Lymphadenopathy was observed in 3 participants dosed in MAD cohorts. The lymphadenopathy was transient and may have been dose/exposure dependent since all 3 events occurred in MAD cohorts (1 participant treated at 7 mg and 2 participants treated at 21 mg dose levels) and after the second infusion of RO7497987. A similar transient lymphadenopathy (Grade 1) was seen in 6 participants who received CDX-301 (25 or 75 μg/kg/day cohorts) and 1 patient that received a single 675 μg dose of GS-3583 in the respective Phase I trials in healthy human volunteers (17, 27).

Clinical studies of GS-3583 administered as single doses in healthy volunteers and as repeated doses up to 20,000 μg in cancer patients (27, 28) have shown a transient dose-dependent increase in cDC expansion (27, 28). Consistent with these prior findings, administration of RO7497987 resulted in dose-dependent expansion of total leukocytes, monocytes, and all major DC subsets, including cDC and pDC with prolonged maintenance of these populations at higher doses. While monocytes demonstrated a dose-dependent increase in cell numbers at the peak of expansion (no sign of C_max_ saturation at the doses tested), DC numbers at the peak expansion plateaued at higher doses (C_max_ saturation around 21 mg), but the levels were maintained for a longer period of time. In the MAD cohorts, a dose level of 7.0 mg demonstrated a cycling effect, while 21 mg dosing resulted in prolonged expansion of DCs and monocytes.

Aberrant FLT3 signaling drives subsets of AML (32) which raises the possibility that FLT3 agonism could promote myeloid leukemogenesis (33). After completing enrollment of this healthy volunteer study, a finding of AML was reported in a Phase I study in patients with advanced solid tumors evaluating the long-acting FLT3 agonist GS-3583. In this study, 1 patient from the cohort assigned to receive GS-3583–20 mg every 2 weeks developed AML as a second primary malignancy during cycle 4 which was reported as treatment-related (28). This patient had received prior anticancer therapies and was reported to be DMT3A positive at baseline (VAF 3%) and found to have NPM1, TET2, and PCLO mutations at the time of diagnosis of AML, suggesting the patient could have had clonal hematopoiesis clone(s) that were undetectable at screening (28, 33). We note that AML was not reported in healthy volunteers who received GS-3583 at doses ranging from 75-625 µg/kg (27), nor in healthy volunteers (17) or patients treated with CDX-301 (24, 26, 34); however, the dose, schedule, and exposure of prior therapies, the age of the volunteer or patient, and other factors could impact susceptibility to any treatment-related risk. The observation of a secondary AML in a patient receiving a FLT3 agonist highlights the risk of leukemogenesis with FLT3 agonism and the importance of developing sensitive screening strategies to exclude patients who may be at elevated risk (33).

Our study contributes to the evolving landscape of FLT3L-based therapies and highlights the potential of RO7497987 to modulate the innate immune compartment. These findings advance our understanding of long acting FLT3 receptor agonism in healthy volunteers. The observed dose-dependent PD effects, including expansion of key DC subsets and monocytes, are consistent with the intended mechanism of action. While no serious safety concerns were identified in this study, the potential for on-target effects, such as secondary malignancy, underscores the importance of risk consideration, careful dose optimization, patient selection, and monitoring.

## Data Availability

For eligible studies qualified researchers may request access to individual patient level clinical data through a data request platform. At the time of writing this request platform is Vivli: https://vivli.org/ourmember/roche/. For up to date details on Roche’s Global Policy on the Sharing of Clinical Information and how to request access to related clinical study documents, see here: https://www.roche.com/innovation/process/clinical-trials/data-sharing. Anonymized records for individual patients across more than one data source external to Roche cannot, and should not, be linked due to a potential increase in risk of patient re-identification. Requests to access the datasets should be directed to https://vivli.org/ourmember/roche/.
