# Supplementary material for: Phase Ia study to evaluate RO7497987, a FLT3L-fragment Fc fusion protein, in healthy volunteers
Source: Front Immunol. 2026 Mar 24;17:1784546. doi: 10.3389/fimmu.2026.1784546 (PMC13055607; doi:10.3389/fimmu.2026.1784546)
Supplement: Supplementary file 1 [file SupplementaryFile1.docx]

Supplementary Table 1. Summary of serum PK parameters in SAD cohorts following IV administration of R07497987.

| **Cohort** | **Dose (mg)** | **N** | **t_max_ (day)^a^** | **C_max_ (μg/mL)** | **AUC_0-21_ (day*μg/mL)** | **AUC_0-inf_ (day*μg/mL)** | **t_1/2_ (days)** | **CL (mL/day)** | **Vss (mL)** |
| --- | --- | --- | --- | --- | --- | --- | --- | --- | --- |
| SAD1 | 0.7 | 6 | 0.0729 (0.0715- 0.0729) | 0.191 (20.4) | 0.354 (25.4) | 0.353 (25.4) | 1.24 (23.9) | 1970 (25.5) | 3600 (20.1) |
| SAD2 | 2.1 | 6 | 0.0729 (0.0729- 0.075) | 0.629 (6.67) | 1.70 (25.4) | 1.69 (25.4) | 1.21 (29) | 1240 (25.4) | 2950 (19.4) |
| SAD3 | 7.0 | 6 | 0.111 (0.0729- 0.229) | 2.17 (29) | 8.58 (39.5) | 8.58 (39.6) | 1.53 (50.8) | 816 (39.6) | 3350 (32.3) |
| SAD4 | 21 | 6 | 0.153 (0.0729- 0.313) | 6.23 (22.4) | 38.0 (26) | 39.5 (30.2) | 4.08 (31.4) | 531 (30.2) | 3950 (12.6) |
| SAD5 | 70 | 6 | 0.0736 (0.0729- 0.313) | 27.6 (22.4) | 173 (19.6) | 203 (21.6) | 12.0 (64.5) | 345 (21.6) | 3810 (17) |
| Concentrations for one participant in SAD2 were normalized by subtracting the baseline from the observed concentrations due to high pre-dose baseline concentrations.  One participant in SAD5 was removed as samples were missing due to early termination.  ^a^T_max_ was reported as median (range). All other parameters were reported as geometric mean (Geometric Mean CV%). | | | | | | | | | |

Supplementary Table 2. Summary of serum PK parameters in MAD cohorts following IV administration of R07497987.

| **Cohort** | **Dose number** | **Dose (mg)** | **N** | **t_max_ after last dose (day)^a^** | **C_max_**  **(μg/mL)** | **AUC_0-21_ (day*μg/mL)**^b^ | **AUC_0-inf_ (day*μg/mL)** | **CL (mL/day)** | **VSS (mL)** | **AR C_max_ (μg/mL)** | **AR AUC (day*μg/mL)** |
| --- | --- | --- | --- | --- | --- | --- | --- | --- | --- | --- | --- |
| MAD1 | 1 | 7.0 | 6 | 0.11 (0.0729-0.313) | 1.94 (17.4) | 7.45 (29.0) | 7.45 (29.1) | 940 (29.1) | 3520  (22.7) | - | - |
| MAD1 | 2 | 7.0 | 6 | 0.0885 (0.0521-0.208) | 2.04 (18.5) | 6.01 (16.4) | NR | NR | NR | 1.05  (15.5) | 0.806  (14.4) |
| MAD2 | 1 | 21 | 6 | 0.146 (0.0736-0.229) | 6.90 (26.5) | 42.7 (34.6) | 46.1 (47.0) | 456  (47.0) | 3430 (19.9) | - | - |
| MAD2 | 2 | 21 | 6 | 0.0524 (0.0521-0.13) | 7.34 (32.4) | 33.1 (59.1) | NR | NR | NR | 1.06  (7.75) | 0.775  (23.8) |
| ^a^T_max_ was reported as median (range). All other parameters were reported as geometric mean (Geometric Mean CV%).  ^b^AUC_0-21_ is calculated relative to the most recent dose, with time of most recent dose set as time = zero. | | | | | | | | | | | |
